# A review of chronic wasting disease (CWD) spread, surveillance, and control in the United States captive cervid industry

**DOI:** 10.1080/19336896.2024.2343220

**Published:** 2024-04-22

**Authors:** Jameson Mori, Nelda Rivera, Jan Novakofski, Nohra Mateus-Pinilla

**Affiliations:** a Illinois Natural History Survey, University of Illinois Urbana-Champaign, Champaign, IL, USA; b Department of Animal Sciences, University of Illinois Urbana-Champaign, Champaign, IL, USA; c Department of Pathobiology, University of Illinois Urbana-Champaign, Urbana, IL, USA; d Department of Natural Resources & Environmental Sciences, University of Illinois Urbana-Champaign, Urbana, IL, USA

**Keywords:** Contact tracing, epidemiology, network, prion, risk assessment, transmission

## Abstract

Chronic wasting disease (CWD) is a fatal prion disease of the family *Cervidae* that circulates in both wild and captive cervid populations. This disease threatens the health and economic viability of the captive cervid industry, which raises cervids in contained spaces for purposes such as hunting and breeding. Given the high transmissibility and long incubation period of CWD, the introduction and propagation of the infectious prion protein within and between captive cervid farms could be devastating to individual facilities and to the industry as a whole. Despite this risk, there does not yet exist a literature review that summarizes the scientific knowledge, to date, about CWD spread, surveillance, or control measures. Our review, which focused on peer reviewed, primary research conducted in the United States, sought to address this need by searching Google Scholar, Scopus, and Web of Science with a five-term keyword string containing terms related to the (1) location, (2) species affected, (3) disease, (4) captive cervid industry, and (5) topic of focus. Between the three databases, there were 190 articles that were selected for further examination. Those articles were then read to determine if they were about CWD spread, surveillance, and/or control in captive cervid facilities. The 22 articles that met these inclusion criteria were evaluated in detail and discussed, with recommendations for future collaborative work between captive cervid owners, government agencies, and researchers. This work will help to address, inform, and mitigate the rising problem of CWD spread and establishment.

## Introduction

Transmissible spongiform encephalopathies (TSEs) are a class of neurodegenerative diseases caused by a misfolded and infectious protein called a *prion* [1]. Prion diseases have been identified in humans, felines, mink, sheep, cattle, camels, and cervids and are characterized by the slow deterioration of neurological tissues, leading to behavioural changes, weight loss, and inevitable death [2–4]. Of these TSEs, the prion disease affecting cervids – chronic wasting disease (CWD) – is the only one known to circulate among both wild and captive animal populations [5]. In the United States (U.S.), a *captive cervid* is defined by the Code of Federal Regulations as ‘all species of deer, elk, moose, and all other members of the family *Cervidae* raised or maintained in captivity for the production of meat and other agricultural products, for sport, or for exhibition, including time such animals are moved interstate; or any wild cervid that is moved interstate, during the period of time from capture until release into the wild’ [6]. Terms used to refer to private land housing captive cervids include – but are not limited to – ‘farm’, ‘ranch’, ‘facility’, and ‘operation’.

CWD was first detected in 1967 in Colorado, U.S. in a captive cervid herd and has since expanded to wild and or/captive cervid populations across the United States, Canada, South Korea, Sweden, Norway, and Finland [5,7]. The disease is transmitted via physical contact and the exchange of bodily fluids (direct contact), or through contact with contaminated materials or equipment (indirect contact) [5]. The disease is highly infectious and slowly progressing, with an incubation period between 16 months [8] and 4 years [9]. Following the onset of symptoms such as weight loss, behavioural changes, and ataxia, animals usually die within four months but can survive up to a year [6]. Affected deer can shed the infectious prion in saliva and faeces as early as nine months post-infection, which is long before the onset of symptoms, as well as in urine later in the disease course [10]. These long incubation periods and shedding of infectious prion into the environment, where it can remain infectious for years [10], pose a particular problem for captive cervid herds, since close quarters and regular contact between animals increases the likelihood of transmission and makes controlling its spread difficult once introduced.

A key element of mitigating disease spread is through identifying cases and removing affected animals as early as possible through diagnostic testing. Currently, only immunohistochemistry (IHC) and enzyme-linked immunosorbent assay (ELISA) are approved for use as diagnostic tests for CWD. New diagnostic tools are also being developed to detect earlier stages of CWD infection, potentially while the animal is still alive [11]. Though all diagnostic tests come with an expense and labour burden [12], detecting CWD is key to formulating prevention strategies to reduce disease spread. The adoption of strict biosecurity protocols to reduce the movement of infected animals and restrict the spread of CWD between and within farms is essential. The United States Department of Agriculture (USDA) outlines biosecurity measures captive cervid facilities should implement: (1) prevent contact between captive and wild cervids, (2) properly dispose of dead animals, (3) practice decontamination of clothing, shoes, equipment, and vehicles on the farm, and (4) avoid exposing captive cervids to biological material from cervids originating outside the farm [13]. However, the severity of the risks associated with not implementing these biosecurity measures are unclear, which complicates the decision-making process for captive cervid owners trying to balance cost with benefit. Another complication is that there is no standard for national data collection, record keeping, individual cervid identification, diagnostic test reporting, or contact tracing for the captive cervid industry. This knowledge gap sharply contrasts with the high need for insights into CWD dynamics in this system that would enable development and implementation of best practices to prevent the movement of disease within and between farms.

**Figure 1.**
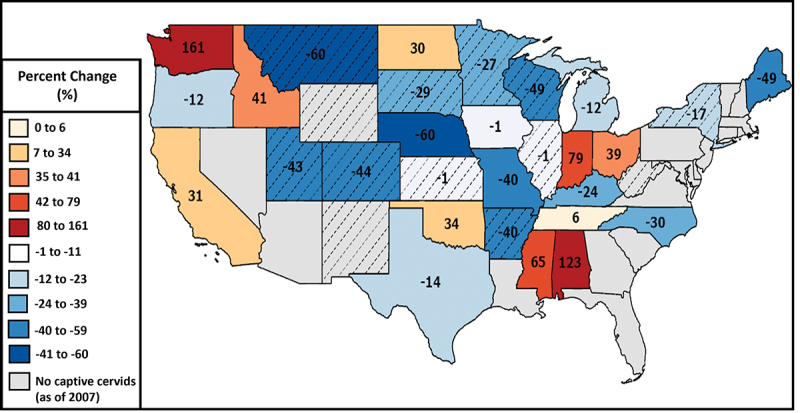
Percent change in the number of captive cervids, by state, between 2002 and 2007, with states reporting cases of chronic wasting disease (CWD) in 2007 indicated with diagonal lines (adapted from Anderson and Chomphosy, 2014).

There are also economic and social impacts associated with CWD in the captive cervid industry [5]. Consumers benefit from a wide range of products and services provided by captive cervid farms, including meat, antlers, breeding stock, urine used by hunters as attractants, and other byproducts [14]. Additionally, some farms keep cervids as pets for owner enjoyment, public exhibition (such as in zoos), and/or educational purposes [15]. The annual estimated economic impact of the cervid industry in Minnesota by 2012 was $17 million, with an estimated 1,287 jobs supported by this industry [14]. The economic losses associated with CWD’s impact on the cervid industry have been estimated for places like Alberta, Canada, and are estimated to range from $12 million (for prevention measures like improving fencing) to hundreds of millions (for compensation by the government for depopulation) [16]. The impact of CWD on the captive cervid industry is not a future problem but a growing and ongoing reality. A review of economic, social, biological, and ecological problems associated with captive cervids cites examples of captive cervid herds having a CWD prevalence of 90% or higher, necessitating depopulation and decontamination procedures that are expensive, demoralizing, and potentially bankrupting [16]. With the financial strain that CWD can place on the captive cervid industry, it is not surprising that a study examining the 2002 and 2007 USDA Census of Agriculture found that the number of captive cervids in a state decreased by 54% on average in states where CWD was present [17] (Figure 1). This study also estimated that the amount of state income lost due to CWD-related factors was around $230 million due to the reduction in captive cervid numbers of around 28% in states with CWD [17].

The consequences of not controlling CWD in captive cervid herds are further made apparent by case-studies that document disease prevalences up to 100% [18–20]. Keane et al. (2008) calculated that the infection rate on their study farm was 20 times higher than what would be observed in wild cervids, likely due to a high number of animals being in close contact for long periods of time and exposed to the same infected animals and environments. These studies highlight what makes the issue of CWD in captive cervids unique compared to the same disease in wild cervids and serve as a reminder why disease mitigation practices need to be developed and adopted nationally.

Studies have been conducted in the United States about the epidemiology of CWD in captive cervid herds [9,19], herd-level factors associated with CWD-positive herd status [21], genetic factors involved with CWD infection and disease progression [22,23], best practices for managing CWD in zoos [24], and the economic impacts of CWD on both wild and captive cervids and the agencies that manage them [12]. Prior publications have raised concerns regarding ethics, animal welfare, public health implications associated with captive cervid facilities [16], and some of these concerns relate specifically to diseases [25]. Though Gerhold and Hickling (2016) did not address CWD, they highlight the translocation of cervids between farms, particularly across state lines, as a significant biosecurity risk. They also point out that a lack of consistency in regulations hampers disease management and control efforts, especially when transactions between farms involve multiple states and supervising agencies [25]. Research on CWD in captive herds conducted in other countries includes work done in Canada on preventing the spread of CWD from captive facilities to local wild cervid populations [26], the epidemiology of outbreaks on captive farms [27,28] and implementation of risk-based surveillance and control efforts in captive facilities [29,30].

While these works address important aspects of CWD in captive cervid herds, no review article has been published that assesses the scientific literature on how CWD moves within and between captive cervid facilities, what is being done, or what could be done, to control this disease spread and expansion in the U.S., even though the need for such information has been demonstrated. To fill this gap, this review gathered the existing peer-reviewed, primary scientific research to identify what is and is not known about the spread, surveillance, and control of CWD in captive cervid facilities in the United States.

## Methods

This review focused on the question of how chronic wasting disease (CWD) impacts captive cervid facilities in the U.S., with a focus on CWD spread, surveillance, and control. A captive cervid facility is defined as a privately owned property on which animals of the family *Cervidae* that fit the Code of Federal Regulations definition of captive cervids are kept [6]. Sources considered for this review were peer-reviewed primary research papers published online in English. We limited our geographic scope to the U.S. to fill the absence of such a review. For the literature search, a keyword search string was entered into the databases Web of Science, Scopus, and Google Scholar on 1 November 2023. The keyword string was as follows: (US OR ‘United States’ OR ‘United States of America’ OR U.S.) AND (‘Cervus canadensis’ OR ‘Odocoileus virginianus’ OR ‘Odocoileus hemionus’ OR ‘Alces alces’ OR ‘Capreolus capreolus’ OR ‘Dama dama’ OR ‘Cervus elaphus’ OR ‘Muntiacus reevesi’ OR ‘Cervus nippon’ OR ‘Rangifer tarandus’ OR ‘Axis axis’ OR Cervid* OR deer OR moose OR elk OR reindeer OR caribou OR wapiti) AND (‘chronic wasting disease’ OR CWD OR ‘spongiform encephalopathy’ OR prion) AND (captive OR farm OR private OR ranch OR facility OR breeder OR captivity) AND (spread OR surveillance OR transmission OR control OR mitigation OR prevent OR manage OR fence OR vaccine OR quarantine OR record OR data OR test). All results from Web of Science and Scopus were examined, as were the Google Scholar results that included the listed keywords in the title or abstract. Duplicated articles were removed.

**Figure 2.**
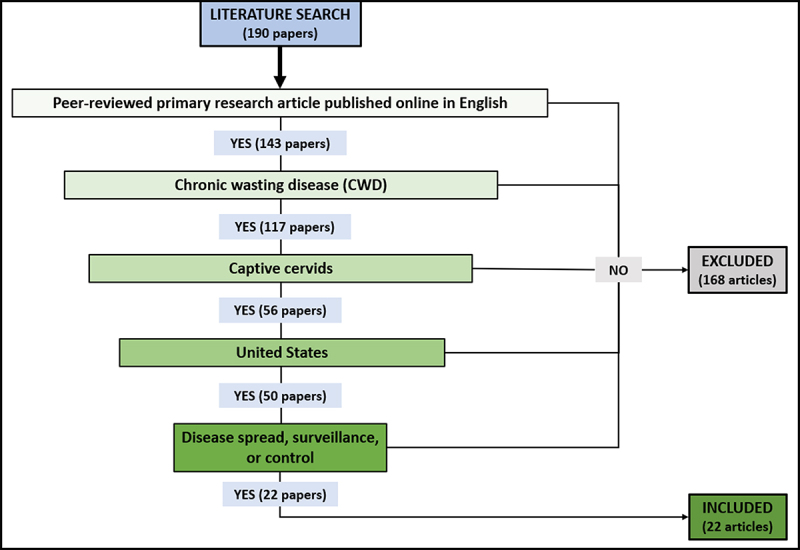
Flowchart outlining literature screening process and outcomes.

Once the set of potentially relevant literature had been collected, the articles were screened using the process outlined in Figure 2. Articles that met all five inclusion criteria were retained. Examples of articles that were excluded for failing to meet one or more criteria are review papers, government reports, or articles published about research in countries other than the U.S. or in wild cervids.

The implications of the included studies for CWD management in captive cervid facilities were assessed, and the limitations and gaps in current knowledge published in peer-reviewed scientific literature were identified.

## Results

The literature review yielded a total of 22 scientific publications that dealt, in some way, with the spread, control, and surveillance of chronic wasting disease (CWD) in captive cervids in the U.S. These papers have been grouped by subject (Table 1), but many address multiple topics and may be referenced in other sections when relevant.

**Table 1.** Peer-reviewed primary research articles addressing the spread, surveillance, and control of chronic wasting disease (CWD) in captive cervid facilities in the U.S. published as of November 2023.

Topic	Analysis	Authors (Year) [Citation]
Diagnostic testing	*‘ … evolution of diagnostic tests for chronic wasting disease … ’*	Haley and Richt (2017) [11]
	*‘ … compare ELISA, IHC, and RT-QuIC … ’*	Holz et al. (2022) [37]
Fencing	*‘ … animal-activated cameras to estimate rates of interaction between wild and farmed deer … ’*	Vercauteren et al. (2007) [34]
	*‘ … effectiveness of a baited electric fence … for reducing fence-line contact between elk.’*	Fischer et al. (2011) [35]
	*‘ … secondary electric fence to prevent contacts in white‐tailed deer … ’*	Khouri et al. (2022) [36]
Genetics	*‘ … infection status and disease stage … correlate our findings with PRNP genotype … ’*	Haley et al. (2019) [22]
	*‘ … prion genotype was strongly linked to progression of prion accumulation in the obex … ’*	Keane et al. (2008) [19]
	*‘ … genome-wide association analyses … ’*	Seabury et al. (2020) [38]
	*‘ … selective breeding program for farmed white-tailed deer … ’*	Haley et al. (2021) [23]
	*‘ … custom Affymetrix Axiom single-nucleotide polymorphism array … ’*	Seabury et al. (2022) [39]
Preventive treatment	*‘ … infection rates … receiving pentosan polysulfate, tannic acid, tetracycline HCl, or no treatment … ’*	Wolfe et al. (2012) [44]
	*‘ … ability of CWD-infected brain material to pass through the gastrointestinal tract of coyotes … ’*	Nichols et al. (2015) [20]
	*‘ … incidence and disease course … while being maintained on sustained-release Cu boluses or unsupplemented … ’*	Wolfe et al. (2020) [45]
Spread and surveillance	*‘ … sustained horizontal transmission of CWD most plausibly explained epidemic dynamics … ’*	Miller et al. (2004) [18]
	*‘ … graph theory in the context of infectious disease epidemics of farmed animals … ’*	Rorres et al. (2018) [31]
	*‘ … time aggregated network analysis … ’*	Makau et al. (2020) [32]
	*‘ … qualitative risk assessment for CWD transmission to cervid farms … ’*	Kincheloe et al. (2021) [33]
	*‘ … risk factors related to multiple pathways of CWD transmission to farmed cervid herds.’*	Schultze et al. (2023) [21]
Vaccination	*‘ … CWD vaccines consisting of cervid prion peptide sequences … ’*	Pilon et al. (2013) [40]
	*‘ … orally inoculated with attenuated Salmonella expressing PrP … ’*	Goñi et al. (2015) [41]
	*‘ … oral immunization of white-tailed deer … ’*	Taschuk et al. (2017) [42]
	*‘ … novel CWD vaccine in elk … targets a YYR disease-specific epitope to induce antibody responses … ’*	Wood et al. (2018) [43]

### Spread and surveillance

#### Risk assessment

**Figure 3.**
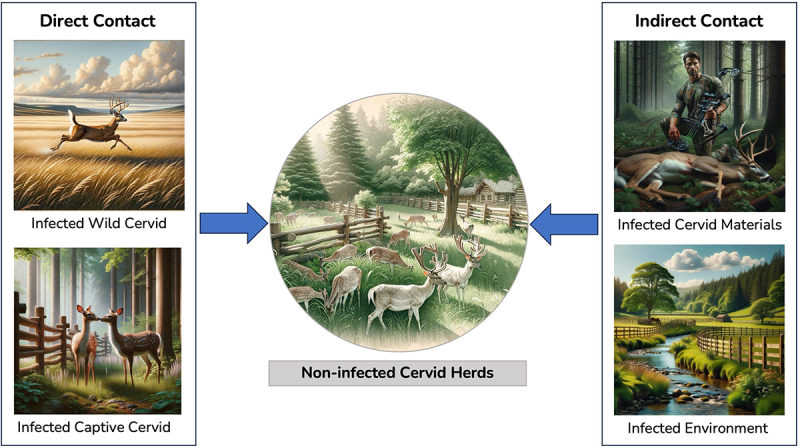
Overview of chronic wasting disease (CWD) transmission routes to uninfected captive cervid herds.

Five articles were found that addressed the spread and surveillance of CWD within and between captive cervid farms. These articles cover topics related to transmission sources and risks for cervid farms, as well as contact networks and tracing tools for CWD in the captive cervid industry. Figure 3, shown below, provides an overview of the different routes through which a noninfected cervid herd could become infected with CWD.

Evaluation of environmental contamination as a risk factor by Miller et al. (2004) confirmed that CWD can be transmitted to naïve cervids indirectly through exposure to an environment contaminated with the pathogenic prion. In this study, the environment was contaminated with the excreta or carcasses of CWD-positive cervids [18]. The experiment involved exposure of uninfected mule deer (*Odocoileus hemionus*) to three scenarios: (1) cohabitation in a paddock with infected deer, (2) habitation of a paddock in which infected carcasses had decomposed 1.8 years prior, or (3) habitation of a paddock in which other infected deer had lived 2.2 years earlier [18]. Transmission of CWD occurred in all three scenarios, demonstrating that direct and indirect transmission pathways both play a role in the propagation of CWD within captive cervid herds [18].

A qualitative risk assessment of CWD transmission to captive cervid farms was conducted by Kincheloe et al.. (2021) and used to identify risks that CWD-positive farms in Minnesota (8 farms) and Wisconsin (26 farms) had experienced [41]. Of the 34 farms, 68% exclusively contained white-tailed deer, and 72% were exclusively breeding farms. Three general types of risk were identified: tissue, secreta/excreta, and environment. Tissue risk involved handling or disposing of potentially contaminated tissues, while risks associated with secretions/excretion involved reproductive procedures or direct physical contact between farmed and wild cervids. Environmental risks included the use of feed, equipment, and other materials that came from a farm that was later determined to have CWD. Risks were stratified into low, moderate, and high, with different levels of uncertainty. Tables 2 and 3 are adapted from the results of this study, with Table 2 showing the breakdown of risk types and levels and Table 3 providing a general view of how many farms experienced at least one low, moderate, or high risk [41].

**Table 2.** Qualitative risk assessment of chronic wasting disease (CWD) for captive cervid facilities in Minnesota and Wisconsin, USA, between 2002 and 2019 [33].

Transmission Pathway	Risk Type	Risk Level	Uncertainty	Exposure Type	Farms
Introduction of farmed cervids	Direct	High	Low	From farm later found to be CWD-positive	35%
		Moderate	High	From farms with no CWD test positive animals in prior 5 years	50%
		Negligible	Low	No record of introductions from other farms in prior 5 years	15%
Contact with wild cervids from farm location <80 km from detection of a CWD-positive wild cervid		High	Low	Farmed cervid escapes/re-entry or wild cervid entry	24%
		Moderate	High	Single perimeter fencing	41%
		Negligible	Low	Double perimeter fencing or >80 km^2^ from wild CWD-positive	35%
Introduction of cervid parts	Indirect	High	Low	From <80 km from CWD-positive wild cervids	6%
		Moderate	High	From other areas	3%
		Negligible	Low	No introductions	91%
Introduction of contaminated fomites or scavenger entrance		High	Low	From CWD-positive farms	?
		Moderate	High	From locations <80 km from CWD-positive wild cervids or farms with no CWD positives	
		Negligible	Low	No indirect contacts

**Table 3.** Risk levels of chronic wasting disease (CWD) experienced by captive cervid farms in Minnesota and Wisconsin, USA, between 2002 and 2019 [33].

State	Risk Level	Farms Experiencing Risk (%)
Minnesota	High	75%
	Moderate	13%
	Negligible	13%
Wisconsin	High	50%
	Moderate	50%
	Negligible	0%

A trend towards an increasing number of CWD-positive captive facilities was observed in this study, though the types and levels of risk varied between farms and over time. It was found that 44% of farms with detected CWD cases did not identify any high transmission risks, such as fence breaches in areas with CWD-affected wild populations or imports from CWD-positive farms, suggesting that moderate risks may be important for CWD introduction, and, that this importance increases as risks compound. Researchers also observed that 80% of farms with CWD-positive animals reported recent imports from other cervid farms. Those farms had not detected any CWD cases but still posed a risk due to non-exhaustive testing [41]. It was also noted that the importance of moderate transmission risk changed over time, with farms that tested positive earlier in the study period identifying far more high transmission risks (85%) than those that tested positive later in the study period (38%) [41]. This could provide evidence for the success of regulatory policies meant to minimize high transmission risks, changes in farm biosecurity practices, or the rising importance of environmental contamination and indirect transmission in CWD-endemic areas. The study emphasized the importance of surveillance and monitoring, as well as diagnostic testing that can accurately detect CWD in the early stages of disease.

A similar paper by Schultze et al.. (2023) evaluated risk factors related to pathways of CWD transmission in Wisconsin, Pennsylvania, and Minnesota, U.S [21]. Seven factors that increased the risk of CWD transmission to farmed cervids were identified: (1) importing cervids from CWD-positive herds, (2) having the captive cervid’s water source be within 0.3 metres of the perimeter fence line, (3) captive cervids being within 5 kilometers of the nearest detected case of CWD in wild cervids, (4) domestic cats having access to captive cervid enclosures or food storage containers, (5) observations of animal scavengers near the perimeter fence, (6) having a perimeter fence that crosses forested areas, and (7) captive cervids sharing a water source with wild cervids [21]. The exact reasons why these factors increased disease transmission risk were not determined experimentally, but proposed explanations include that these factors increased direct or indirect contact with infected cervids or environments.

#### Deer movement and contact tracing

The identification of sources of risk for CWD transmission is essential for managing the disease on an individual farm, but there is also the issue of disease spreading between multiple farms. In order to improve contact tracing for CWD, a study by Rorres et al.. (2018) applied graph theory to data collected as part of Pennsylvania’s CWD Herd Monitoring Program – in which captive cervid farms are required to participate – to determine contact networks between farms [39]. Records of 5269 separate within-state deer transfers between 681 unique farms from 14 October 2003, through 19 June 2011, were obtained [39]. The graph theory approach then took the movement data and created a network of contacts between farms so that a particular farm was determined to be connected to another if at least one deer was transferred from it within 24 hours [39]. Most of these shipments (69%) were within 50 miles of the original facility, though there were exceptions with movements up to 300 miles [39]. Analysis was also done to quantify the relationships between ‘first-case farms’ – the first farm in a chain of potential infection to have a diagnosed case of CWD – and farms that the facility had had contact with prior to and after case detection to gain an understanding of what the effects of CWD appearing in these contact networks might be [39].

**Figure 4.**
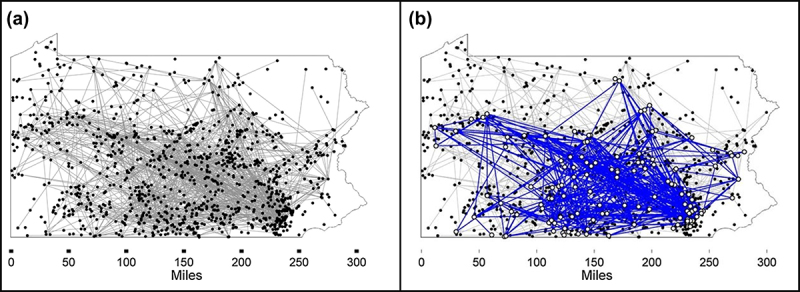
Contact networks between captive cervid farms in Pennsylvania, USA (Panel A), with the largest strongly connected component (139 farms) shown in blue (Panel B), between 2003 and 2011 [39].

The results of the graph analysis revealed multiple subnetworks of between-farm connections that formed *strongly connected components (SCC)* in the graph [39]. An SCC is a network of contacts between farms such that if any farm within it was infected, it could infect any other farm that was also within that SCC, which has major implications for disease surveillance and control [39]. Sixteen such SCC’s were observed in the graph, with 12 of these consisting of 2 farms each, 2 with three farms, 1 with four farms, and 1 including 139 farms [39]. Figure 4 shows the maps of the network of connections between captive cervid farms in Pennsylvania, U.S., with Figure 4(a) showing the whole network and Figure 4(b) showing the largest SCC (139 farms) in blue.

The scale of the largest SCC, and of the smaller but still significant SCCs, determines the scale of contact tracing and surveillance that would be necessary to properly control an outbreak of CWD on farms that are part of these networks. The more highly connected the farms are, the more labour intensive the mitigation efforts will be. For this reason, the authors of the study suggested that these SCCs should be broken up and the movement of cervids restricted to only between carefully chosen pairs of facilities [39]. This study emphasized the complex nature of connections within the industry of cervid farming and the importance of thorough record-keeping at the farm and state levels.

Another study done by Makau et al. (2020) addressed a similar question in Minnesota using time aggregated network analysis on 1221 cervid transfers between 432 facilities recorded between 2013 and 2018 [40]. The Minnesota Board of Animal Health requires captive cervid producers to report all animal movements, which provided the information necessary to conduct a k-test analysis on the captive farm network to determine how well it reflected possible pathways of CWD transmission. The study also examined how far CWD could spread through the network, with consideration of temporal effects. The study found that 48.2% of the cervid movements in Minnesota were within the state and that movement frequency greatly increased in September and October, prior to the breeding and hunting seasons [40]. These transfers included 11,298 cervids, mainly white-tailed deer (61.4%) and elk (33.1%) [40]. Unlike the study in Pennsylvania [39], which found many strongly connected components, the Minnesota study found only a giant weakly connected component that included 90.3% of farms [40]. The observation that movements between specific pairs of farms were rarely repeated likely contributed to the weak connections within this network.

While the typical cervid farm in Minnesota had only weak connections to others in this network, analysis of data specifically from CWD-positive farms revealed that these facilities were more likely to be connected than would be explained by random chance, with documented cervid movements between 2 of the 3 CWD-affected farms [40]. The study also found that these CWD-positive farms received transfers from 2 to 12 other farms and transferred their deer to between 2 and 7 farms. Due to the long incubation period of CWD [8,9] and the unreliability of diagnostic testing in the earlier stages of the disease [11,31], the potential for so much movement of deer from infected facilities prior to first detection points to the need for more thorough and accurate testing and extensive record keeping for contact tracing. The results of this study emphasized the need for comprehensive record-keeping for all states because of the high number of inter-state deer transfers, and the authors suggested that surveillance be heightened during increased animal movements in the Fall [40].

#### Control

##### Fencing

Three manuscripts dealt with the issue of fencing around captive cervid facilities and the potential for contact between wild and captive cervids along this fence line. One study observed the rate of contact between wild and farmed deer and noted only two direct contacts [32], while two studies examining the use of electrified secondary fencing documented a higher number of contacts and the cessation of those contacts when a secondary electric fence was used [33,34]. These studies reinforce a finding in both Kincheloe et al. (2021) and Schultze et al. (2023), which identified perimeter fencing as a potential weak point for disease transmission and stressed the importance of ensuring that fencing is sufficient to reduce contact between wild and captive cervids.

##### Genetics

One approach to handling CWD in captive cervid farms has been to breed for genetic resistance to CWD. This genetic resistance is due to the different variants of the prion protein, with some variants being more susceptible to misfolding than others [22]. Prion genotype has also been strongly associated with the rate of prion accumulation in the obex [19]. Some studies have demonstrated that this variation in prion proteins is a heritable trait that could be bred for [23,35,36], with the study by Haley et al. (2021) formulating a selective breeding programme that captive cervid farmers could follow to increase the CWD resistance of their herd. Further work needs to be done to determine the full role of genetics in disease susceptibility and progression, and how the interaction between genetics and disease transmission plays out in a herd.

##### Vaccination

Four studies explored the possibility of developing a vaccine for chronic wasting disease. A study published in 2013 tested a vaccine made of cervid prion proteins associated with slowed disease onset on mule deer (*Odocoileus hemionus*) but found that the deer still became infected when challenged with the pathogenic prion [42]. A 2015 study looked at a modified *Salmonella* vaccine engineered to express the cervid prion protein and found that while most of the deer still became infected with CWD, the vaccine significantly prolonged the incubation period by around 300 days [43]. One vaccinated deer did remain uninfected, even after oral challenge with CWD-infected tissue [43].

A team of researchers also developed a blueprint for vaccine development that was shown to be generally successful in inducing an antibody response to the pathogenic prion protein [44]. This vaccine did not induce a sufficient immune response to be successful as a vaccine, but the study provided a basis for future work in CWD and other wildlife and livestock diseases [44].

In 2018, a vaccine targeting the misfolded prion protein was tested in captive elk (*Cervus canadensis*) that were kept in an environment contaminated with CWD [45]. Instead of slowing down or preventing CWD disease, it was found that vaccinated elk had a faster disease onset than unvaccinated elk [45]. The reason for this faster onset is unclear. Despite the general lack of success in developing a CWD vaccine so far, research towards that goal is ongoing.

##### Preventive treatment

Outside of vaccination, other preventive treatments for CWD have been tested in cervids. One study compared infection rates in mule deer that had been treated with either tannic acid, tetracycline HCl, pentose polysulphate, to deer that had received no treatment [37]. None of the treatments were deemed effective, though the authors note that the rapid disease course of the infected animals indicates the initial challenge dose may have been too high [37]. Another study looked at dietary supplementation with copper, but this treatment had no impact on CWD susceptibility or survival rates [38].

A study that did not work directly with captive cervids, but bears mentioning, analysed water and soil samples from CWD positive and negative cervid facilities, as well as areas where CWD is endemic to wildlife to quantify magnesium and copper [20]. This study found higher levels of both elements at CWD negative sites, and feeding laboratory mice more magnesium and copper increased their survival times after inoculation with CWD [20]. More work needs to be done to confirm the link between these two elements and CWD disease onset and progression.

##### Diagnostic testing

**Figure 5.**
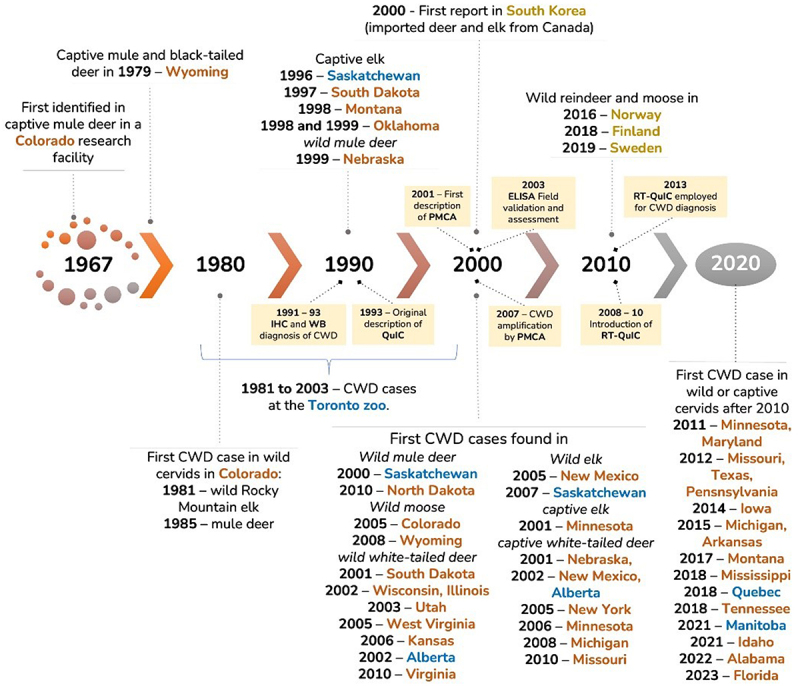
Timeline of chronic wasting disease (CWD) in North America and diagnostic test development.

Since diagnostic testing for CWD is not unique to captive cervids, and has been thoroughly addressed in other literature reviews, we will only provide a brief overview of the timeline of diagnostic test development (Figure 5) and general details of each test with references.

Caption: Immunohistochemistry (IHC), enzyme-linked immunosorbent assay (ELISA), western blot (WB), protein misfolding cyclic amplification (PMCA), and real-time quaking-induced conversion (RT-QuIC). States, provinces, and countries are indicated as follows: United States of America (orange), Canada (blue), and outside North America (yellow). Timeline adapted from Haley and Richt (2017), Hibler et al., 2003, CWD Alliance, and Rivera et al. (2019).

The official diagnostic test (the ‘gold standard’) for CWD is immunohistochemistry (IHC), which is applied to lymphoid and neurological tissues to identify the misfolded protein [11,31]. IHC involves breaking down and staining the proteins in the tissue sample to detect whether any proteins remain intact. Only the misfolded prion protein can undergo the digestion process without breaking down, so detection of intact proteins indicates the animal was positive for CWD [11]. Cell damage caused by the misfolded prion is also visible, if present, and the source of the tissue sample can be confirmed through inspection of the sample, with these factors contributing to the use of IHC as the established diagnostic test for CWD [11]. The only laboratory test other than IHC officially approved for diagnosing CWD is enzyme-linked immunoassay (ELISA), with all positive results from ELISA confirmed with IHC using the same tissue. Morphologic diagnostic options include examination of brain and lymphatic tissue under a microscope to identify the characteristic spongiform appearance of CWD-infected brain tissue and observe the accumulated infections prion protein in lymphatic tissues. Additional diagnostic tools like Western blot, protein misfolding cyclic amplification (PMCA), and real-time quaking-induced conversion (RT-QuIC) [11,31] have been used in research studies and continued to be developed.

Discussions surrounding diagnostic testing for CWD in captive cervid herds revolve around balancing test accuracy, cost, and ease of administration. Many, if not all, studies of biosecurity in the captive cervid industry emphasize the importance of an antemortem diagnostic screening tool to facilitate limiting the movement of infected animals in the early stages of the disease. A study conducted on antemortem testing of rectal biopsies from a captive elk herd using RT-QuIC and IHC demonstrated two major lessons associated with this issue. First, the study showed that RT-QuIC identified more CWD-positive animals than IHC did and avoided factors known to influence the sensitivity of IHC, providing some evidence for the value of these applications [22]. Second, the disease burden in the study herd became so high that the owners were no longer able to maintain the herd, emphasizing the importance of having a tool available that allows for the isolation of suspects in early stages of infection, and facilitates prevention of whole herd infection [22].

## Discussion

### Potential gaps in knowledge

Of the 22 studies that met the inclusion criteria for this review, 5 addressed CWD spread and surveillance in captive cervid facilities while the remaining 17 focused on different aspects of disease control. This is a relatively low number of studies given the rapid expansion of this industry. The fact that there are only 22 studies examining CWD spread, surveillance, and/or control in the captive cervid industry points to a significant gap in knowledge about how this disease affects the captive cervid industry. This is in stark contrast to the risks highlighted in case studies of CWD in these herds, which document high prevalence and extreme measures such as depopulation [19]. There are also over three times more studies on CWD control than on its spread and surveillance, likely reflecting the fact that most states do not require record keeping or reporting from these captive cervid farms [46,47], and so the data needed to conduct these studies is unavailable to researchers. The studies included in this review analysed data from states that had more rigorous government oversight than is typically observed across the U.S [48], and the insights gained from those studies show the importance of gathering and sharing this data so that our collective understanding of this disease can be expanded to develop practical and effective management recommendations. These observations can be distilled into the two major conclusions of this review: (1) detailed data needs to be collected for all cervid movements and farm activities and (2) this data needs to be made available to researchers and government officials.

### Key points to consider

**Figure 6.**
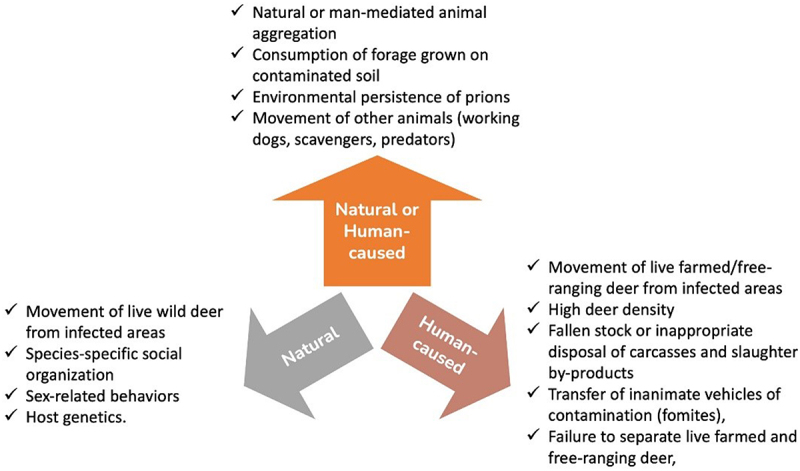
Summary of identified risk factors based on their biological plausibility to spread CWD (adapted from EFSA 2019 [49]).

Though the studies included in this review cover a wide range of topics, time spans, and geographical locations, they agree on several points. One main consensus is that the captive cervid industry is a complex network and that the level of complexity is influenced by the level of government regulation and how long the industry has been established in that state. Despite the heterogeneity of the captive cervid industry when compared within or between states, it was consistently noted that CWD-affected farms are more closely related than they would be by random chance, indicating that CWD is spreading through these networks. Spread between farms was concluded to be due to direct contact (animal to animal) via transfer of infected animals, but indirect/environmental transmission within a farm was also implicated, especially when the disease had been long established in a herd. In addition to the risks associated with importing animals or animal tissues from farms or areas that may be CWD-positive, other risk factors for CWD spread included inadequate fencing, sharing equipment or other materials with another farm that may be CWD-positive, direct or indirect contact between wild and captive cervids (i.e., physical contact through fencing or shared water sources), and potential transfer of infectious materials through the movements of other animals, like scavengers or domestic cats (Figure 6). A list of risk factors based on their biological plausibility was developed by the European Food Safety Authority (EFSA) using decades of studies in the U.S [49], which are summarized in Figure 6.

The studies identified in this review paper emphasized the importance of diagnostic testing of animals, particularly before moving animals between farms, to detect infections early and limit disease transmission and prion shedding into the environment. When the question was raised, all studies also acknowledged the potential benefits of a vaccine or other preventative treatment for CWD, though there are none, to date, that are effective and available.

There were some aspects that these studies did not come to a consensus on, such as which cervids to test and when. Makau et al. (2021) suggests increasing diagnostic testing in the Fall to reflect the higher number of cervid movements, but no other suggestions like this are made. The two studies on the connectivity between captive cervid farms also disagreed on the level of connectivity, with Rorres et al. (2018) finding high connectivity between farms in Pennsylvania, U.S. and Makau et al. (2021) finding low connectivity between farms in Minnesota, U.S. This is likely due to differences between how both states regulate captive cervids and how long the industry had been in place at the time of the study. Both studies do agree that better managing the connectedness of the farms could improve the biosecurity of the whole system and prevent CWD-positive animals from being transferred to multiple farms before detection.

### The future of CWD management and surveillance for captive herds

**Figure 7.**
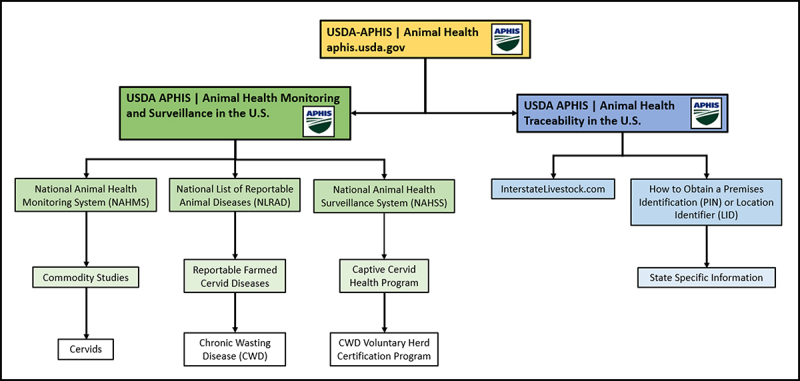
National-level regulation for chronic wasting disease in the U.S., as of 2023.

There remain many significant unknowns about how CWD affects the captive cervid industry. The question of how CWD could be regulated at the national level in the U.S. is an open and divisive one. Figure 7 shows how CWD is regulated at the national level as of 2023. A more detailed diagram providing a full breakdown of the government agencies regulating the captive cervid industry, with sources, is provided in the Supplementary Materials.

Guidelines for diagnostic testing to optimize disease detection, efficiency, and economic burden have yet to be developed for captive cervids, despite testing being a critical tool in disease management. The exact ways wild and captive cervids interact, either directly or through a shared environment, are also still being determined [50]. The role of fomites in CWD transmission on or between farms also remains unclear. There are also currently no recommendations for how to decontaminate land that has housed CWD-infected cervids, though a laboratory study by Sohn et al. (2019) demonstrated that application of sodium hydroxide reduced the infectivity of prions in soil. However, the safety and practicality of this, or other, measures remain unknown. A study on the impact of humic acids on CWD prions in the soil did demonstrate that these acids break down the misfolded protein and reduce its infectivity [51], making this a promising topic of future research for the possible of use of humic acids, or other soil compounds, for decontamination efforts.

Overall, awareness of and mitigation efforts for chronic wasting disease will benefit captive cervids, their owners, and all individuals who interact with the industry and maintain its biosecurity and economic success. A survey of stakeholder (e.g., captive cervid owners and hunters) risk perception of CWD, wildlife agencies, and agriculture professionals in New York State identified apparent differences among groups [52]. While New York State biologists and other wildlife and agricultural professionals (not employed by the state) thought CWD risks associated with captive cervids were high, captive cervid owners thought risks for captive and wild cervids were low [52]. Similar results were identified related to any pathways involving inter-state import of live cervids, where all groups except captive cervid owners gave a high ranking of risks to inter-state import. It is important to note that New York State successfully managed the 2005 CWD outbreak -as no recurrence of CWD was reported as of 2015 when the survey was conducted, so perceptions may be different in other states with CWD. The survey provides a clear example of how vital it is to understand risk perceptions to facilitate effective disease prevention and management strategies. Education and communication are also essential to avoid misinformation and encourage cooperation [52].

### Best practices for CWD management on captive cervid farms

From the information gathered from the literature, a set of best practices for moving and maintaining captive cervids was assembled based on the available evidence.

1. Care should be taken when importing cervids from other farms. Find out if those farms are certified CWD free, or if they are using diagnostic testing on their animals. If the CWD status of the imported animals in unknown, quarantine and test them upon arrival before allowing contact with the existing herd. Ideally, animals should be quarantined for at least a year and repeatedly tested for the disease.
2. Don’t share equipment or materials with other farms.
3. Keep all water and food sources separate from outside the farm (i.e. using troughs for water instead of a stream).
4. Utilize double perimeter fencing to prevent contact between wildlife and captive cervids.
5. Maintain detailed records of all cervid movements into and out of the farm, including fence breaches with influx of wild deer or escape of captive deer.

## Conclusion

This review highlights the current gaps in our knowledge about CWD spread, surveillance, and control in the U.S. captive cervid industry. The included studies emphasize the importance of data collection and sharing, CWD mitigation measures, and the potential serious repercussions that CWD has for the captive cervid industry. To maintain and increase the success of this industry nationwide, it is necessary to promote open communication between captive cervid farmers, regulatory agencies, and wildlife disease researchers. This communication will better enable the sharing of information and increase the overall understanding of CWD and the captive cervid industry, helping to foster success in the future.
