# Correction

**DOI:** 10.1080/19336896.2024.2359752

**Published:** 2024-05-30

Correction: PRION

Article title: *A review of chronic wasting disease (CWD) spread, surveillance, and control in the United States captive cervid industry*

Authors: Jameson Mori, Nelda Rivera, Jan Novakofski, and Nohra Mateus-Pinilla

Journal: *PRION*

DOI: https://doi.org/10.1080/19336896.2024.2343220

The author of the article has requested a correction in the in-text citation, and the reference citations have been rectified in the original article.

Figure 2

**Figure 2.**
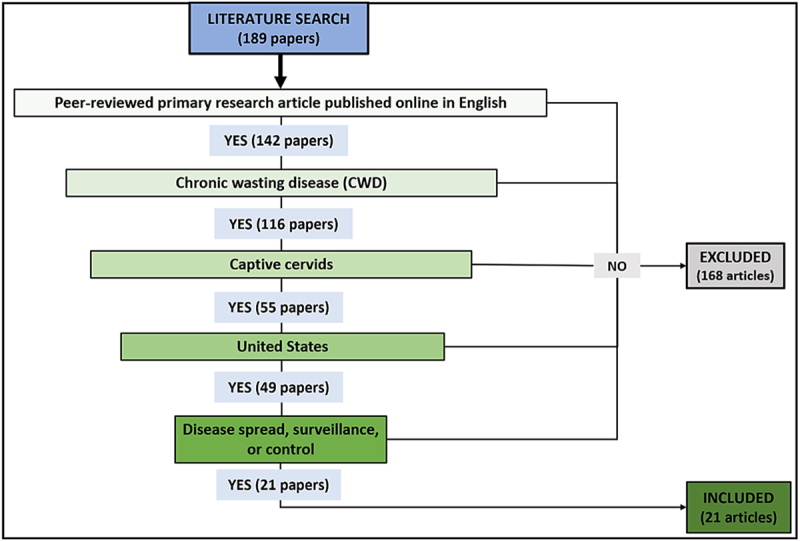

has been substituted with the following figure:

In the section “Spread and Surveillance,” the subsection content titled “Genetics” has been modified as follows:

One approach to handling CWD in captive cervid farms has been to breed for genetic resistance to CWD. This genetic resistance is primarily due to the different variants of the prion protein, with some variants being more susceptible to misfolding than others [22]. Prion genotype has also been strongly associated with the rate of prion accumulation in the obex [19]. Some studies have demonstrated that this variation in the prion protein is a heritable trait [22,34,35], opening up the possibility that these genetic tools could be used to selectively breed for lower CWD susceptibility in captive cervid herds. Further work needs to be done to determine the full role of genetics in disease susceptibility and progression, and how the interaction between genetics and disease transmission plays out in a herd.

Additionally, the values of the articles and publications in the abstract and text have been rectified.

All the aforementioned changes have been implemented, and the article has been republished.

